# Genome-wide identification and expression analysis of the coronatine-insensitive 1 (*COI1*) gene family in response to biotic and abiotic stresses in *Saccharum*

**DOI:** 10.1186/s12864-021-08255-0

**Published:** 2022-01-08

**Authors:** Tingting Sun, Yintian Meng, Guangli Cen, Aoyin Feng, Weihua Su, Yanling Chen, Chuihuai You, Youxiong Que, Yachun Su

**Affiliations:** 1 Key Laboratory of Sugarcane Biology and Genetic Breeding, Ministry of Agriculture and Rural Affairs, College of Agriculture, Fujian Agriculture and Forestry University, Fuzhou, 350002 Fujian China; 2 College of Life Sciences, Fujian Agriculture and Forestry University, Fuzhou, 350002 Fujian China; 3 Key Laboratory of Genetics, Breeding and Multiple Utilization of Crops, Ministry of Education, College of Agriculture, Fujian Agriculture and Forestry University, Fuzhou, 350002 Fujian China

**Keywords:** Sugarcane, *COI1* gene family, Whole genome analysis, Biotic and abiotic stresses, Expression analysis

## Abstract

**Background:**

The coronatine insensitive 1 (*COI1*) gene is the core member of jasmonate signaling pathway, which is closely related to plant biotic and abiotic resistance. However, there have been no reports on *COI1* in sugarcane (*Sacharum* spp.). Hence, systematically investigating the characteristics of the *COI1* multigene family in sugarcane can provide a means to study and manipulate the jasmonic acid signaling pathway.

**Results:**

A total of 156 COI1 proteins were obtained from the genomes of 19 land plants, while none were obtained from five algae species. A phylogenetic tree demonstrated that these COI1 proteins were classified into four groups, while 31 proteins of SsCOI1 from *Saccharum spontaneum*, SbCOI1 from *Sorghum bicolor*, and ShCOI1 from *Saccharum* spp. hybrid cultivar R570 clustered into three groups. Synteny analysis and duplication patterns revealed that *COI1* genes expanded through various genome replication events and could have experienced strong purifying selective pressure during evolution in *S. spontaneum*, *S. bicolor*, and R570. An investigation of *cis*-acting elements suggests that *COI1* genes may be involved in plant growth and development and response to various stresses. Expression analysis implied that 21 *SsCOI1* genes were constitutively expressed, and had positive responses to drought, cold, and *Sporisorium scitamineum* stresses with different expression patterns. Among them, seven *SsCOI1* haplotype genes may play different roles in response to methyl jasmonate. Furthermore, the *ShCOI1–4*, *ShCOI1–5*, and *ShCOI1–6* genes were cloned from *Saccharum* spp. hybrid cultivar ROC22. Real-time quantitative PCR (RT-qPCR) analysis demonstrated that these three *ShCOI1* genes had divergent expression profiles in response to salicylic acid, abscisic acid, polyethylene glycol, cold, and *S. scitamineum*.

**Conclusions:**

These results suggest that *COI1* genes may act in sugarcane growth, development, and response to various stresses via different regulatory mechanisms, which laying a foundation for the functional identification of the sugarcane *COI1* gene.

**Supplementary Information:**

The online version contains supplementary material available at 10.1186/s12864-021-08255-0.

## Background

As signal molecules, jasmonics, including jasmonic acid (JA) and its cyclopentanous derivatives, play an important role in plant growth [1], development [2, 3], and response to biotic and abiotic stresses [3, 4]. According to previous reports, the core members of the JA-signaling pathway, including SCF^COI1^ (SCF, SKP1 + Cdc53/cullin+Rbx1 + F-box; COI1, coronatine insensitive 1) E3 ubiquitin ligase, jasmonate ZIM-domain (JAZ) repressor proteins, and myelocytomatosis2 (MYC2) transcription factor, have been defined as the COI1/JAZs/MYC2 module [5, 6].

In recent years, the mechanistic details of the JA signal transduction pathway and its regulatory network have gradually been revealed [7–9]. Research on jasmonics mainly focuses on their metabolism and signal transduction [9], their interaction with other hormones [10–12], and the responses of JA signals to pathogenic bacteria or pests [10, 13, 14]. JA content is low in plants under normal growth conditions, and JAZ inhibits the expression of JA-response genes through direct interaction with transcription factors such as MYC2. JA-mediated responses are therefore repressed by JAZ proteins [6, 15]. In response to stresses, such as that caused by insect feeding or pathogen infection, JA is accumulated rapidly, and jasmonoyl-l-isoleucine (JA-Ile) is formed under the action of jasmonic acid-amido synthetase (JAR1) [6, 16]. The increase of JA-Ile levels promotes the interaction between the JAZ repressor protein and the F-box protein encoded by *COI1*, making JAZs ubiquitinate and degrade via the 26S proteasome pathway, releasing DNA-binding transcription factors (such as MYC2) and inducing the expression of JA response genes [6, 15]. In addition, the deletion of the *COI1* locus or its functionally deficient mutations can lead to the elimination or weakness of plant responses to JA [13, 17]. These results illustrate the importance of COI1 in JA signal transduction.

It has been reported that *COI1* is not only involved in plant developmental processes, such as leaf senescence [18, 19], seed maturation [13], flowering [20], male fertility [21], anthocyanin formation [22], and root growth [22, 23], but also plays a part during various physiological processes in the plant defense response to insect attack and pathogen infection [13, 24, 25]. *COI1* belongs to a multigene family. There are one F-box domain and 16 leucine-rich repeats (LRR) in COI1 protein [26, 27]. The F-box protein participates in the formation of the SCF^COI1^ E3 ubiquitin ligase complex involved in the ubiquitin-dependent proteolytic pathway [28], and regulates the expression of JA-responsive genes [28, 29]. As a JA receptor, COI1 protein is maintained at a protein level essential for proper biological functions during plant development and defense, which is strictly regulated by the dynamic balance of SCF^COI1^-mediated stabilization and 26S proteasome pathway-mediated degradation [30, 31]. To date, 35 COI1-dependent JA-regulated proteins have been identified in *Arabidopsis thaliana* [19]. Among them, rubisco activase (RCA), which is correlated with JA-induced leaf senescence, can be down-regulated by JA in a COI1-dependent manner [19]. In addition, *Arabidopsis coi1* mutants were observed to be male-sterile [17], apical dominance defective (*coi1–37*) [32], susceptible to pests and bacterial pathogens [17], insensitive to JA [17], and lacking the expression of JA-induced proteins [33]. *Arabidopsis coi1–1* mutant plants were infertile and showed a stay-green phenotype under dark-induced senescence conditions, but those phenotypes could be rescued in mutants overexpressing *35S:OsCOI1a* or *35S:OsCOI1b* due to the fact that the JA signaling insensitivity of *coi1–1* mutants was complemented. This finding suggests that *coi1* plays a key role in leaf senescence and fertility [18, 34]. In a study by Huang et al. [22], amino acid changes in COI1 could significantly attenuate its function, not only in regulating JA-inhibited root growth and JA-induced anthocyanin accumulation, but also in JA-mediated plant response to inoculation with the pathogen *Pst* DC3000. However, different mutations in the *COI1* gene have distinct effects on *COI1* function in regulating male fertility. *GhCOI1* silencing in *Gladiolus hybridus* impaired inducible defense and increased susceptibility to the necrotrophic pathogenic fungus *Alternaria brassicicola* [24]. The above findings have provided evidence of the importance of the *COI1* gene during the processes of plant growth and development, as well as defense responses. However, there have been no reports on the *COI1* gene in sugarcane (*Sacharum* spp.). Hence, a systematic investigation of the characteristics of the *COI1* multigene family in sugarcane should provide an efficient basis for the study and manipulation of the JA signaling pathway.

Sugarcane is an important sugar and biofuel crop in the world [35, 36]. However, various stresses, such as pathogens, low temperatures, and drought, seriously restrict the healthy development of the sugarcane industry [37]. Due to the complex genetic background and long growth period of sugarcane, genetic engineering has great advantages in the cultivation of resistant sugarcane varieties compared to traditional cross breeding [38]. Therefore, the discovery of resistance candidate genes is of great significance. In the present study, first, 21 *SsCOI1*, three *ShCOI1*, and seven *SbCOI1* genes were identified from the genomes of the sugarcane-related wild species *Saccharum spontaneum* [36], the sugarcane*-*related cultivated species *Saccharum* spp. hybrid cultivar R570 [38], and the sugarcane proximal species *Sorghum bicolor* [39], respectively. Second, the protein physicochemical properties, chromosome location, evolutionary relationship, protein motif, gene structure, *cis*-acting elements, tissue-specific expression, and expression profiles of the *COI1* gene family under methyl jasmonate (MeJA), cold, drought, and *Sporisorium scitamineum* stresses were analyzed [40–42]. Third, the full-length sequences of three *ShCOI1* genes (*ShCOI1–4*, *ShCOI1–5*, and *ShCOI1–6*) were isolated from *Saccharum* spp. hybrid cultivar ROC22 using a homologous cloning method. In addition, the real-time quantitative PCR (RT-qPCR) technique was used to analyze the gene expression patterns of *ShCOI1–4*, *ShCOI1–5*, and *ShCOI1–6* under cold, drought, salicylic acid (SA), abscisic acid (ABA), and *S. scitamineum* stresses [42–44]. This study aims to uncover and identify the *COI1* gene family in sugarcane, understand their sequence characteristics and gene expression patterns, and thus provide candidate gene resources for sugarcane resistance molecular breeding.

## Results

### Identification, classification, and phylogenetic analysis of *COI1* gene family

**Fig. 1:**
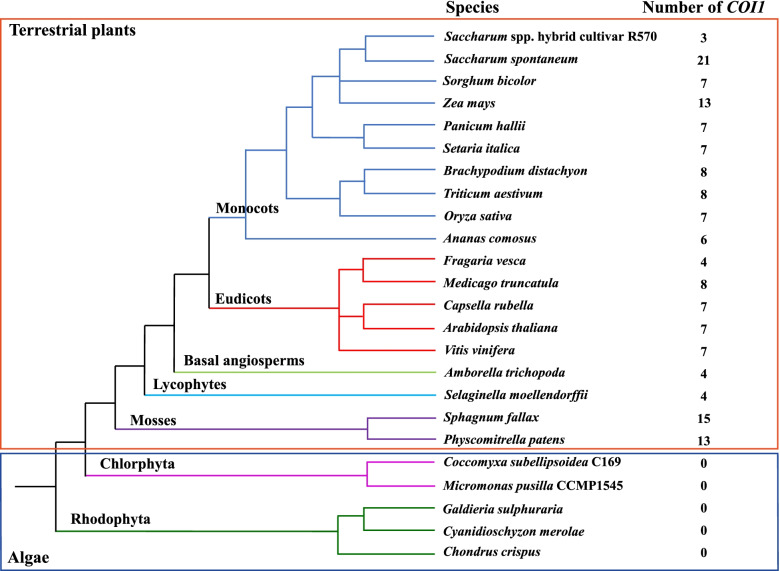
The number of *COI1* homologue genes and the evolutionary relationship of 24 species. These 24 species used in this study belonged to seven lineages (Monocots, Eudicots, Basal angiosperms, Lycophytes, Mosses, Chlorphyta, and Rhodophyta) that are derived from terrestrial plants and algae

A total of 156 COI1 proteins were obtained from 19 sequenced plant species among five lineages, including 33 COI1s in five eudicots (eight in *Medicago truncatula*; seven in *A. thaliana*, *Capsella rubella*, and *Vitis vinifera*; and four in *Fragaria vesca*), 87 COI1s in 10 monocots (21 in *S. spontaneum*; 13 in *Zea mays*; eight in *Brachypodium distachyon* and *Triticum aestivum*; seven in *Oryza sativa*, *Panicum hallii*, *Setaria italica*, and *S. bicolor*; six in *Ananas comosus*; and three in R570), four COI1s in one basal angiosperm (*Amborella trichopoda*), 28 COI1s in two mosses (15 in *Sphagnum fallax* and 13 in *Physcomitrella patens*), and four COI1s in one lycophyte (*Selaginella moellendorffii*). However, no COI1 was identified in five algae plants that belonged to Rhodophyta (*Chondrus crispus*, *Cyanidioschyzon merolae*, and *Galdieria sulphuraria*) and Chlorophyta (*Coccomyxa subellipsoidea* C169 and *Micromonas pusilla* CCMP1545) (Fig. 1 and Supplemental Table S1).

**Fig. 2:**
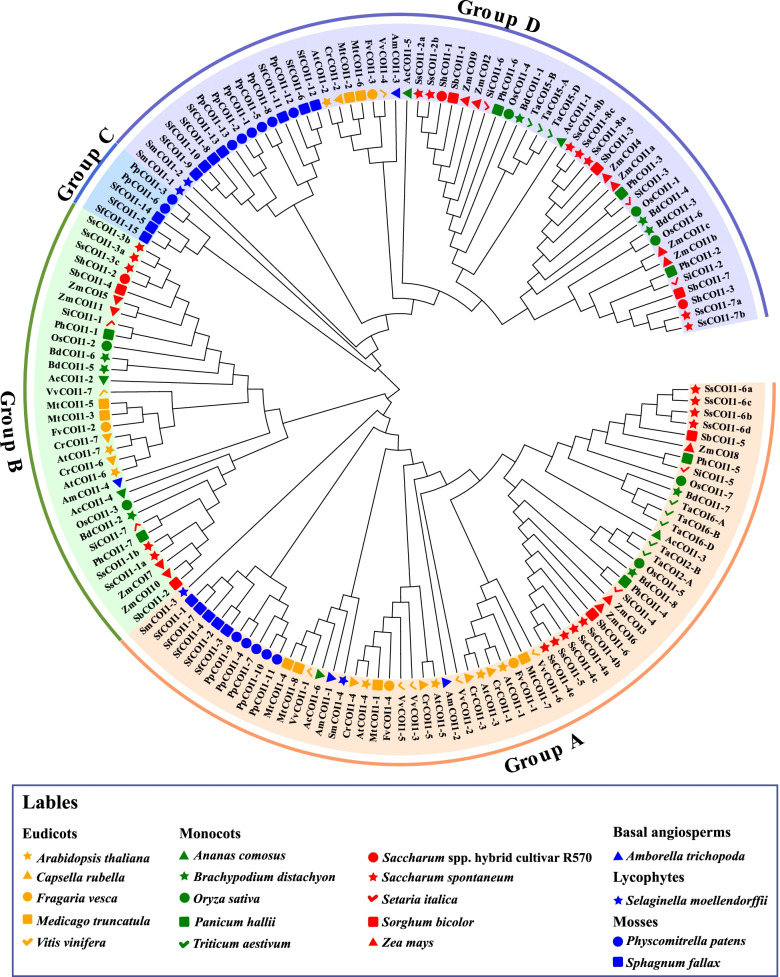
Phylogenetic analysis and classification of 156 COI1 proteins in 19 plant species. The phylogenetic tree was constructed using the maximum likelihood method (JTT + G model, complete deletion, and 1000 bootstrap replicates) using MEGA 6.60 based on the full-length sequences of COI1 proteins. Four colored arcs indicated four groups of COI1 proteins. The different colored shapes corresponded to various species were shown in labels. All the accession numbers of COI1 proteins were listed in Supplemental Table S1

On the basis of the topology of phylogenetic trees and the conserved amino acid sites of F-box and JAZ-binding sites on COI1 proteins (Supplemental Fig. S1 and Supplemental Table S2) [45], 156 COI1 proteins were classified into four groups (group A, group B, group C, and group D) (Fig. 2). Among them, *COI1* genes from the same lineage, such as mosses, monocots, and eudicots, tended to be clustered to the same branch in group A, group B, and group D, and only COI1 proteins from mosses were clustered in group C. In detail, group A contained nine SsCOI1s (SsCOI1–4a, −4b, −4c, −4e, −5, −6a, −6b, −6c, and −6d) and two SbCOI1s (SbCOI1–5 and SbCOI1–6). Group B included five SsCOI1s (SsCOI1–1a, −1b, −3a, −3b, and −3c), two SbCOI1s (SbCOI1–2 and SbCOI1–4), and ShCOI1–2. There were seven SsCOI1s (SsCOI1–2a, −2b, −7a, −7b, −8a, −8b, and −8c), three SbCOI1s (SbCOI1–1, SbCOI1–3, and SbCOI1–7), and two ShCOI1s (ShCOI1–1 and ShCOI1–3) in group D.

### Characteristics of the *COI1* gene family in *S. bicolor*, R570, and *S. spontaneum*

As shown in Supplemental Table S3, the number of amino acids of 31 COI1s (including 21 SsCOI1s, seven SbCOI1s, and three ShCOI1s) was 434–665, and their corresponding molecular weights (MWs) ranged from 47.94 to 73.14 kDa. The predicted isoelectric point (*p*I) values of 31 COI1s varied from 5.25 to 8.40. The results of the instability index suggested that 30 of 31 COI1s were unstable proteins (instability index > 40), and the grand average of hydropathicity (GRAVY) showed that 22 of 31 COI1s were hydrophilic proteins (GRAVY < 0). There were no signal peptides or transmembrane structures in these COI1 proteins, suggesting that they were all non-secreted proteins (Supplemental Table S3). Moreover, among 31 COI1 proteins, nine were predicted to be located in the cytoplasm and nucleus, four in the cytoplasm, and 18 in the nucleus (Supplemental Table S3). For the secondary structures of these proteins encoded by the *SsCOI1s*, *ShCOI1s*, and *SbCOI1s*, alpha helixes (45.41–53.46%) and random coils (28.80–37.89%) were the main components, the extended chain (10.90–14.60%) was secondary, and beta turns (3.20–5.84%) accounted for the smallest proportion (Supplemental Table S4).

### Conserved motifs and gene structures of the *COI1* gene family

**Fig. 3:**
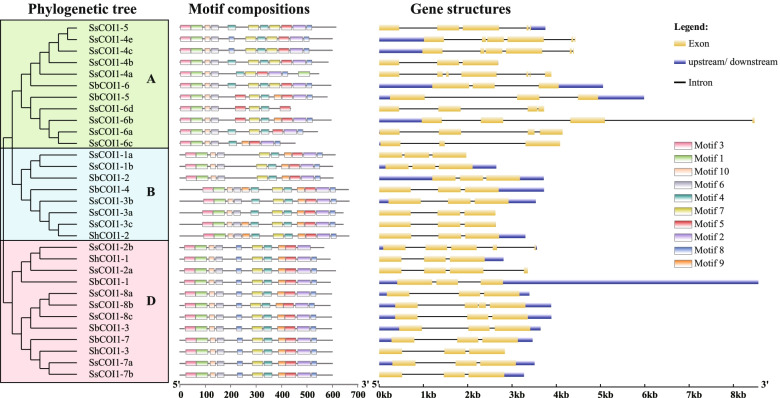
Protein motif and gene structure of *SbCOI1*, *ShCOI1*, and *SsCOI1* genes. *SbCOI1*, *ShCOI1*, and *SsCOI1* represented the *COI1* gene in *Sorghum bicolor*, *Saccharum* spp. hybrid cultivar R570, and *S. spontaneum*, respectively*.* The clustering tree on the left side of the figure was constructed using the maximum likelihood method (JTT + G model, complete deletion, and 1000 bootstrap replicates) using MEGA 6.60, and three evolutionary groups (group A, group B, and group D) were shown in different colors. Motifs were identified using Multiple Em for Motif Elicitation. Motif 1–10 was represented by different colored squares. Details of the individual motifs were shown in Supplementary Table S6. The yellow box, blue box, and black line in the gene structures represented exons, untranslated regions (UTR), and introns, respectively. The sizes of motifs, exons, and introns can be estimated using the scale below

Homology analysis showed that the amino acid sequence similarity among 31 COI1 proteins ranged from 27.00 to 100.00% (Supplemental Table S5). A phylogenetic tree (Fig. 3) demonstrated that the protein sequences of 21 SsCOI1s, seven SbCOI1s, and three ShCOI1s were divided into three groups (groups A, B, and D), which was consistent with the above classification in Fig. 2. The number of conserved motifs in 31 COI1 proteins varied from eight to 12, and motifs 1–7 and motif 10 were included in all of these COI1 protein sequences (Fig. 3). Motif 3, motif 2, and motif 1 represented an F-box_5 domain (pfam18791), a typical LRR sequence domain, and a transport inhibitor response 1 protein domain (cl40087), respectively (Supplemental Table S6). However, several motifs were specific in subgroup members. For example, motif 9 was present in all members of group B and group D, but only in three members of group A. Group D members had two motif 8 and one motif 4, except for SsCOI1–2b. In contrast, motifs in the members of group A were relatively irregular. Likewise, among 11 members of group A, four had two motif 4, five had two motif 7, three had motif 5, and three had two motif 8. These results indicate that motif 1, motif 3, motif 6, and motif 10 are relatively conserved in the evolution of the *COI1* gene family. The number of introns contained in the *COI1* gene family of *S. bicolor*, *S. spontaneum* and R570 ranged from two to six (Fig. 3). The numbers of group B had two introns. In group D, except for SsCOI1–2b, SsCOI1–2a, and SsCOI1–8b, all the other nine members had two introns. However, the gene structures of group A members were irregular, with intron numbers ranging from two to six (Fig. 3).

### Chromosomal location, duplication events, and synteny analysis of *COI1* gene family

Chromosome mapping showed that 21 *SsCOI1* genes were unevenly distributed on 18 of 32 *S. spontaneum* chromosomes (Supplemental Fig. S2 and Supplemental Table S1). Among them, chromosomes Ss1A, Ss4B and Ss5C had two *SsCOI1* genes, and each of the remaining 15 chromosomes had one *SsCOI1* gene. Three *ShCOI1* genes were evenly distributed on chromosomes Sh01, Sh04, and Sh09 among 10 R570 chromosomes (Supplemental Fig. S2). However, *SbCOI1–1* and *SbCOI1–*2 were distributed on Sb01, and the other five *SbCOI1* genes were evenly distributed on chromosomes Sb03, Sb05, Sb05, Sb06, and Sb09 of *S. bicolor* (Supplemental Fig. S2).

**Fig. 4:**
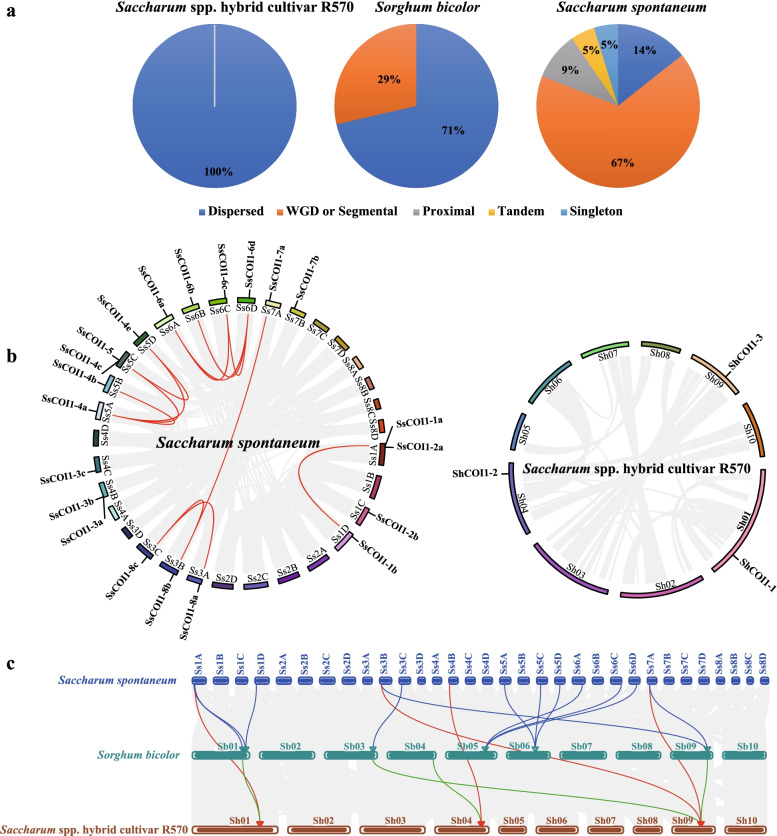
Expansion mechanisms and synteny analysis of *COI1* gene family. **a** Expansion mechanisms of *COI1* gene family in *Sorghum bicolor*, *Saccharum* spp. hybrid cultivar R570, and *S. spontaneum*. The numbers in the pie chart represented the effects of WGD or segmental (whole-genome duplication/segmental duplication), Dispersed (dispersed duplication), Proximal (proximal duplication), Tandem (tandem duplication), and Singleton (single copy). **b** Synteny analysis of *COI1* genes in *S. spontaneum* and R570. **c** Synteny analysis of *COI1* genes in *S. bicolor*, R570, and *S. spontaneum*. All replicated genes in the genome were represented by gray lines in figures **b** and **c**; the red lines in figure **b** represented the replicated *SsCOI1* genes; the red lines in figure **c** represented the homologous *COI1* gene pairs in *S. spontaneum* and R570; the green lines represented the homologous *COI1* gene pairs in *S. bicolor* and R570; and the blue lines represented the homologous *COI1* gene pairs in *S. spontaneum* and *S. bicolor*. “Ss”, “Sh”, and “Sb” represented the name of each chromosome in *S. bicolor*, R570, and *S. spontaneum*, respectively. The detailed information was shown in Supplemental Table S7 and Table S8

To explore explored the expansion mechanisms, the gene types of *COI1* in *S. spontaneum*, R570 and *S. bicolor*, including singleton, dispersed, proximal, tandem, and whole-genome duplication (WGD)/segmental duplications, were analyzed (Fig. 4a and Supplemental Table S7). In 21 *SsCOI1* genes, 14 WGD/segmental (66.67%), three dispersed (14.29%), two proximal repeat genes (9.52%), one tandem (4.76%) duplication, and one singleton gene (4.76%) were found. Interestingly, all three *ShCOI1* genes in R570 had dispersed duplications. Among seven *SbCOI1* genes, five were detected as dispersed genes (71.43%), and two were WGD/segmental duplications (28.57%) (Fig. 4a and Supplemental Table S7). Therefore, it can be speculated that the *SsCOI1* gene family mainly expanded through WGD or segmental duplication events, and the dispersed duplications appear to be the main expansion mechanisms for the *SbCOI1* gene family and *ShCOI1* gene family.

The gene collinearity among *S. spontaneum*, R570, and *S. bicolor* was analyzed to investigate the evolutionary mechanism of the *COI1* gene family (Fig. 4b, c and Supplemental Table S8). In *S. spontaneum*, 12 pairs (14 genes) of collinearity relationships of 21 *SsCOI1s* were observed (Fig. 4b and Supplemental Table S8). Furthermore, all of these 14 genes had WGD/segmental duplications, including 11 pairs (13 *SsCOI1* genes) of homoeologous genes that were distributed in different chromosomes. In R570, there was no collinear relationship among three *ShCOI1* genes in R570 (Fig. 4b and Supplemental Table S8). As shown in Fig. 4c*,* there were 12 orthologous pairs between *S. spontaneum* and *S. bicolor*, four between *S. spontaneum* and R570, and four between *S. bicolor* and R570. The nonsynonymous (Ka)/synonymous (Ks) ratios of all duplicated *COI1* genes in *S. bicolor*, R570, and *S. spontaneum* were < 1, indicating that the *COI1* gene family might have experienced strong purifying selective pressure during evolution (Supplemental Table S8).

### *Cis*-acting elements in the promoter regions of the *COI1* gene family

**Fig. 5:**
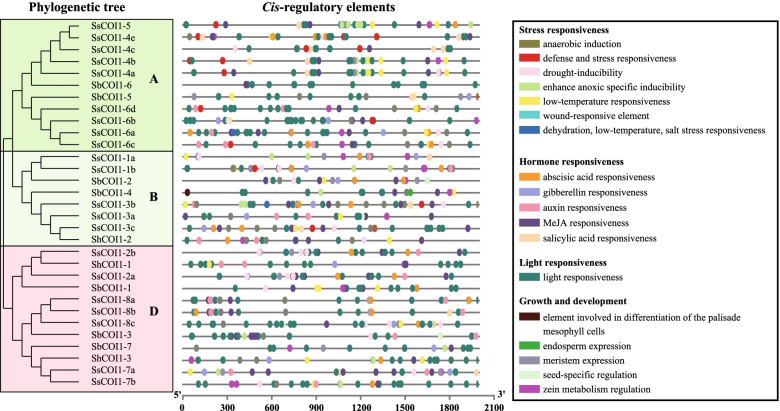
Promoter *cis*-regulatory element analysis of *SbCOI1*, *ShCOI1*, and *SsCOI1* gene family. *SsCOI1*, *ShCOI1*, and *SbCOI1* represented *COI1* genes from *Saccharum spontaneum*, *Saccharum* spp. hybrid cultivar R570, and *Sorghum bicolor*. Different colors boxes in *cis*-regulatory elements correspond to different elements. The clustering tree on the left side of the figure was constructed using the maximum likelihood method (JTT + G model, complete deletion, and 1000 bootstrap replicates), and different color on the phylogenetic tree represented different groups of COI1. The detailed information about promoter *cis*-regulatory elements of *SbCOI1*, *ShCOI1*, and *SsCOI1* genes was shown in Supplemental Table S9

The *cis*-acting regulatory elements in the promoters of *COI1* genes were predicted to assist the gene function elaboration. There were many core elements in the promoter sequences of 31 *COI1s*, which were involved in stress responsiveness, hormone responsiveness, light responsiveness, and growth and development (Fig. 5 and Supplemental Table S9). The light, ABA and MeJA response elements were the most numerous in the *COI1* gene promoter regions, followed by anaerobic induction, drought-inducibility, and low-temperature responsiveness. Among them, light response elements were observed in all *COI1* promoter regions. In 31 *COI1* promoter regions, 30 *COI1s* contained MeJA response elements (CGTCA-motif and TGACG-motif), except for *SbCOI1–5*, 26 (83.87%) contained abscisic acid response elements (ABRE), and 24 (77.42%) contained anaerobic induction elements (ARE), while 22 (70.97%) contained drought-inducibility (MBS) and low-temperature response elements (LTR). In addition, the numbers of *COI1* promoter regions that contained auxin (IAA), gibberellin (GA), and SA response elements, were 16, 18, and 12, respectively. The existence of these functional elements indicates that *COI1* genes may participate in the induction of multiple stress responses and thus play a role in sugarcane defense against various environmental stresses.

### Tissue expression profiles of *COI1* genes in sugarcane

**Fig. 6:**
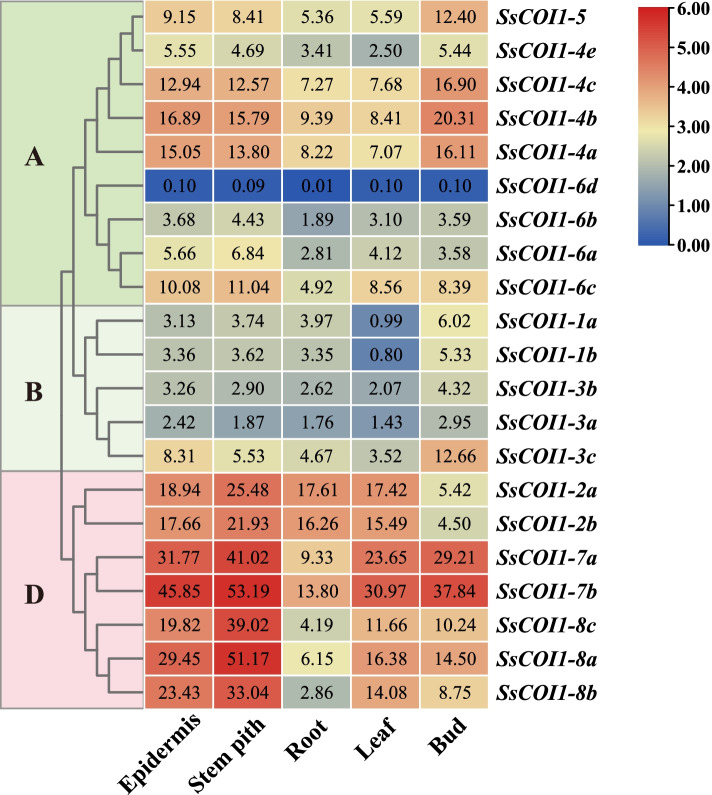
Expression pattern of *SsCOI1* genes in different sugarcane tissues of mature ROC22. Epidermis, Stem pith, Root, Leaf, and Bud represented different tissues of mature ROC22. The fragments per kilobase of transcript per million mapped (FPKM) shown in the box represented the *SsCOI1s* expression levels. The color bar represented the normalized values (log_2_ FPKM). The clustering tree on the left side of the figure was constructed using the maximum likelihood method (JTT + G model, complete deletion, and 1000 bootstrap replicates) by MEGA 6.60, and different colors and letters (**A**, **B**, and **D**) on the phylogenetic tree represented three groups of *SsCOI1s*

The results of transcriptome sequencing (RNA-seq) showed that 21 *SsCOI1* genes were constitutively expressed in the root, bud, leaf, stem pith, and epidermal tissues of sugarcane cultivar ROC22 (*Saccharum* spp. hybrid), but with various expression levels (Fig. 6). Among them, *SsCOI1–6d* had low expression in all tissues, and the expression levels of *SsCOI1–1a*, *−1b*, *−3a*, *−3b*, and *−3c* (clustered into group B) were the highest in the bud and the lowest in the leaf. *SsCOI1–2a* and *SsCOI1–2b* had the lowest expression levels in the bud and the highest in the stem pith. The expression levels of *SsCOI1–6a*, *−6b*, *−6c*, *−7a*, *−7b*, *−8a*, *−8b*, and *−8c* were the highest in the stem pith and the lowest in the root. The highest expression levels of *SsCOI1–4a*, −*4b*, −*4c*, and −*5* were observed in bud tissues, followed by those in the epidermis, stem pith, root, and leaf. There were different expression patterns between *SsCOI1–4e* and its duplicated gene, *SsCOI1–4c*. It was noteworthy that the *SsCOI1* genes that clustered to group D showed a higher expression level compared with the other group in general. These results suggest that *SsCOI1* genes may play a role in sugarcane growth and development, but with different function modes.

### Expression profiles of *COI1* genes under cold and drought treatments

**Fig. 7:**
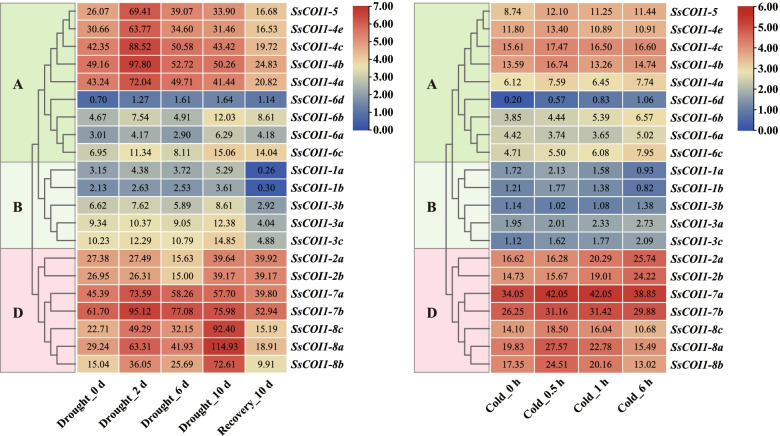
Expression profiles of *SsCOI1s* under drought and cold stresses. The trimmed mean of M-values (TMM) shown in the box represented the expression levels of *SsCOI1s*. The color bar represented the normalized values (log_2_ TMM). The clustering tree on the left side of the figure was constructed using the maximum likelihood method (JTT + G model, complete deletion, and 1000 bootstrap replicates) by MEGA 6.60, and different colors and letters (**A**, **B**, and **D**) on the phylogenetic tree represented three groups of *SsCOI1s*. Drought_0 d, Drought_2 d, Drought_6 d, Drought_10 d, Recovery_10 d, Cold_0 h, Cold_0.5 h, Cold_1 h, Cold_6 h represented the time that sugarcane cultivated under drought or cold treatments

Due to the fact that the drought-inducibility (MBS) and low-temperature response elements (LTR) were observed in most of the promoter sequences of *SsCOI1s*, their expression profiles under drought and cold stresses were analyzed to further investigate the function of *SsCOI1* genes. As shown in Fig. 7, *SsCOI1* responded to both drought and cold stresses, but with different expression patterns.

Under drought stress, *SsCOI1–1a*, *−1b*, *−3a*, *−3b*, *−3c*, *−8a*, *−8b*, and *−8c* had the highest transcription levels at 10 d and the lowest expression levels after water recovery. The expression levels of *SsCOI1–2a* and *SsCOI1–2b* were down-regulated at 6 d, but up-regulated at 10 d and after water recovery. Compared with the control, the expression levels of *SsCOI1–4a*, *−4b*, *−4c*, *−4e*, *−5*, *−7a*, and *−7b* were increased and reached a single peak at 2 d, but decreased to the lowest after 10 d water recovery. The transcription levels of *SsCOI1–6a*, *−6b*, *−6c*, and *−6d* were increased and had the highest levels at 10 d after drought treatment, but all of them were decreased after water recovery. Under cold stress, the expression levels of all *SsCOI1s* were elevated. For instance, *SsCOI1–4b* and *SsCOI1–4e* showed the highest expression levels at 0.5 h and the lowest expression levels at 1 h. The expression levels of *SsCOI1–1a*, *−1b*, *−4c*, *−5*, *−8a*, *−8b*, and *−8c* were all enhanced to a peak at 0.5 h and then decreased. The transcription levels of *SsCOI1–2b*, *−3a, −3c*, *−4a*, *−6b*, *−6c*, and *−6d* were continuously up-regulated from 0 to 6 h after cold treatment, while *SsCOI1–2a*, *−3b*, and *−6a* decreased first and then increased and showed the highest levels at 6 h. In addition, *SsCOI1–7a* and *SsCOI1–7b* were up-regulated at 0.5 h and remained stable at 1 h after cold treatment.

### Expression profiles of *COI1* genes in response to sugarcane smut pathogen infection

**Fig. 8:**
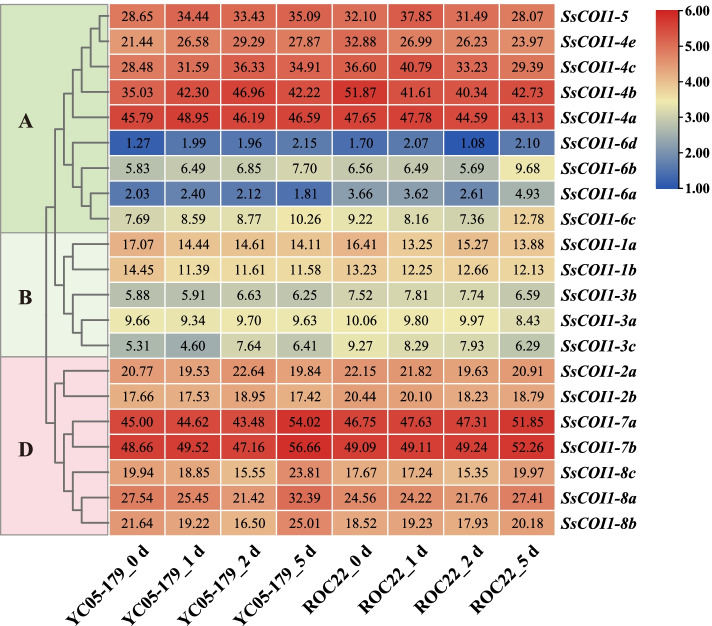
Expression patterns of *SsCOI1s* in the interaction between different sugarcane genotypes and *Sporisorium scitamineum*. The fragments per kilobase of transcript per million mapped (FPKM) shown in the box represented the *SsCOI1s* expression levels. The color bar represented the normalized values (log_2_ FPKM). The clustering tree on the left side of the figure was constructed using the maximum likelihood method (JTT + G model, complete deletion, and 1000 bootstrap replicates) by MEGA 6.60, and different colors and letters (**A**, **B**, and **D**) on the phylogenetic tree represented three groups of *SsCOI1s*. YC05–179_ 0 d/ 1 d/2 d/5 d and ROC22_0 d/1 d/2 d/5 d represented the sugarcane smut-resistant cultivar YC05–179 and smut-susceptible cultivar ROC22 under *S. scitamineum* treatment for 0 d, 1 d, 2 d and 5 d, respectively

To study the function of *SsCOI1s* in response to smut pathogen infection, the gene expression patterns during the interaction between two different sugarcane genotypes and *S. scitamineum* were analyzed. As shown in Fig. 8, in sugarcane smut-resistant *Saccharum* spp. hybrid cultivar YC05–179, *SsCOI1–3a* had no significant expression change while *SsCOI1–1a* and *SsCOI1–1b* were down-regulated. *SsCOI1–4a* and *SsCOI1–6b* were up-regulated and reached the highest expression levels at 1 day post-inoculation (dpi), while *SsCOI1–2a*, *−2b*, *−3b*, *−3c*, *−4b*, *−4c*, and *−4e* were up-regulated and reached a single peak at 2 dpi. The transcription levels of *SsCOI1–5*, *−6b*, *−6c*, and *−6d* were increased to a peak at 5 dpi. In addition, the expression levels of *SsCOI1–7a*, *−7b*, *−8a*, *−8b*, and *−8c* were decreased at 1–2 dpi and then increased at 5 dpi. In sugarcane smut-susceptible *Saccharum* spp. hybrid cultivar ROC22, the transcription levels of *SsCOI1–1a*, *−1b*, *−2a*, *−2b*, *−3a*, *−3c*, *−4a*, *−4b*, and *−4e* were inhibited after smut pathogen inoculation. The expression levels of *SsCOI1–3b*, *−4c*, and *−5* were up-regulated at 1 dpi, and then down-regulated from 2 dpi to 5 dpi. The expression levels of *SsCOI1–6a*, *−6b*, *−6c*, *−6d*, *−8a*, −*8b*, and *−8c* were decreased and reached the lowest point at 2 dpi, and then increased at 5 dpi. The expression levels of *SsCOI1–7a* and *SsCOI1–7b* remained stable from 1 dpi to 2 dpi, and then were up-regulated at 5 dpi.

These results indicated that all 21 members of the *SsCOI1* gene family could be induced during the interaction between sugarcane and smut pathogen, and these allele genes showed similar expression patterns.

### Expression profiles of *COI1* genes under MeJA treatment via RT-qPCR

**Fig. 9:**
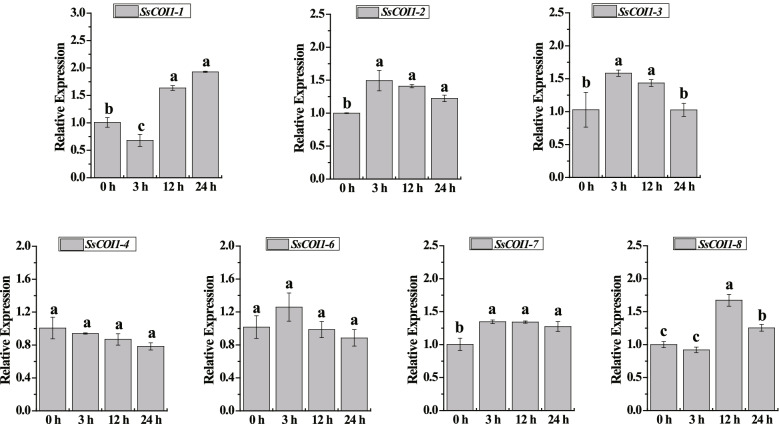
Expression profiles of *SsCOI1s* under MeJA treatment via RT-qPCR. MeJA, 100 μM jasmonic acid. Glyceraldehyde-3-phosphate dehydrogenase (*GAPDH*) was used as a reference gene to normalize the expression data. The 2^−ΔΔCT^ method was applied to obtain the relative expression levels. All data was shown as mean ± standard error (*n* = 3). Different letters representing significant differences were assessed by Duncan test (*p < 0.05)

The *COI1* gene is the core member of the JA signaling pathway. To understand the expression pattern of the *COI1* gene in response to JA, the expression levels of seven *SsCOI1* haplotype genes under MeJA stress were evaluated via RT-qPCR (Fig. 9). After MeJA treatment, the expression levels of *SsCOI1–4* and *SsCOI1–6* (the members of group A) remained unchanged from 0 h to 24 h. The expression levels of the *SsCOI1–1* gene (a member of group B) decreased significantly at 3 h, but increased significantly at 12 h and remained at a high level at 24 h, at levels 1.62- and 1.92-fold that of the control, respectively. The expression levels of *SsCOI1–3* (a member of group B) were increased remarkably at 3 h and 12 h, at levels 1.54- and 1.40- fold higher than the control, and returned to the control level at 24 h. The transcription levels of *SsCOI1–2* and *SsCOI1–7* (members of group D) were up-regulated at 3 h and then stayed at a relatively stable level. For the *SsCOI1–8* gene (a member of group D), its expression levels increased with a single peak at 12 h, which was 1.67-fold higher than the control. The above results show that various *SsCOI1* genes might play different roles in response to MeJA.

### Cloning and sequence analysis of three *COI1* genes in the sugarcane cultivar ROC22

To further understand the functions of *COI1* genes in a sugarcane cultivar, three candidate *COI1* genes, *SsCOI1–4b* (clustered into subgroup A), *SsCOI1–1b* (clustered into group B), and *SsCOI1–3b* (clustered into group B), were cloned from ROC22 and termed as *ShCOI1–4*, *ShCOI1–5*, and *ShCOI1–6*, respectively. The full lengths of the cDNA of the *ShCOI1–4*, *ShCOI1–5*, and *ShCOI1–6* genes were 1953 bp, 2152 bp, and 2322 bp, respectively, with 592, 600, and 661 encoding amino acids, respectively. The amino acid sequence similarities of *ShCOI1–4*, *ShCOI1–5*, and *ShCOI1–6* to *SsCOI1–4b*, *SsCOI1–1b*, and *SsCOI1–3b*, respectively, were 99.50, 99.70, and 99.2%, respectively (Supplemental Table S5). All three ShCOI1 proteins contained relatively conservative F-box domains, Transp_inhibit domains, and AMN1 domains (leucine-rich repeat protein) (Supplemental Fig. S1). Furthermore, compared with ZmCOI1a (GRMZM2G125411), ZmCOI1b (GRMZM2G151536), ZmCOI1c (GRMZM2G353209), and ZmCOI2 (GRMZM2G079112), three ShCOI1 proteins possessed only 4–5 of 16 key amino acid residues (asterisks and sites 1–4 in Supplemental Fig. S1) that are supposed to be the binding sites of JA-Ile or JAZ proteins, suggesting that there may be functional differentiation among these three ShCOI1s and ZmCOIs.

### Gene expression patterns of *ShCOI1–4*, *ShCOI1–5*, and *ShCOI1–6* in response to different abiotic stresses via RT-qPCR

**Fig. 10:**
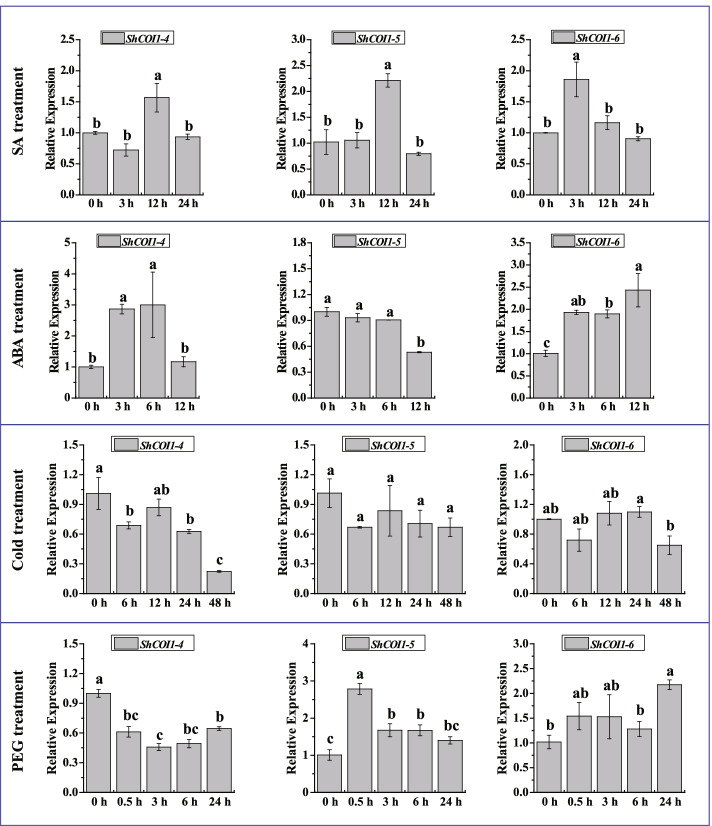
Expression patterns of *ShCOI1–4*, *ShCOI1–5*, and *ShCOI1–6* in response to different abiotic stresses via RT-qPCR. ABA, 100 μM abscisic acid; SA, 5 mM salicylic acid; Cold, 4 °C low temperature; PEG, 25% polyethylene glycol 8000. Glyceraldehyde-3-phosphate dehydrogenase (*GAPDH*) was used as a reference gene. The 2^−ΔΔCT^ method was applied to obtain the relative expression levels. All data was shown as mean ± standard error (*n* = 3). Different letters representing significant differences was assessed by Duncan test (*p < 0.05)

According to previous reports, JA and SA are mostly related to plant resistance to pathogen infection [11, 46], and ABA is mostly associated with abiotic stress [47, 48]. In this study, RT-qPCR was used to analyze the expression levels of *ShCOI1–4*, *ShCOI1–5*, and *ShCOI1–6* in ROC22 under SA, ABA, cold (4 °C), and drought (polyethylene glycol, PEG) treatments (Fig. 10). Under ABA stress, the transcripts of *ShCOI1–4* were up-regulated by 2.86- and 2.99-fold at 3 h and 6 h, respectively, and returned to the control levels at 12 h. After SA treatment, the expression levels of *ShCOI1–4* increased with a single peak at 12 h that was 1.56-fold higher than the control. Under cold and drought stresses, compared to the control, the transcripts of *ShCOI1–4* were decreased. Under SA treatment, the transcripts of the *ShCOI1–5* gene increased with a single peak at 12 h that was 2.17-fold higher than the control. For ABA treatment, the expression levels of *ShCOI1–5* remained unchanged at 0–6 h and were reduced at 12 h. In addition, the transcription levels of *ShCOI1–5* were increased remarkably at 0.5 h, 3 h, and 6 h to levels 2.77-, 1.66-, and 1.66-fold higher than the control under drought stress, respectively, but the transcription levels remained unchanged under cold stress. The expression levels of the *ShCOI1–6* gene were stable under cold stress, but increased under SA, ABA, and drought stresses. After SA treatment, the expression level of *ShCOI1–6* was increased remarkably at 3 h to a level 1.86-fold higher than the control. Under ABA treatment, the expression levels of *ShCOI1–6* were increased at 3–12 h, and peaked at 12 h. The expression level of *ShCOI1–6* was significantly increased at 2.14-fold higher than the control at 24 h after drought treatment.

Based on these findings, it is speculated that *ShCOI1–4*, *ShCOI1–5*, and *ShCOI1–6* actively respond to biotic and abiotic stresses in plants via different signal pathways.

### Gene expression patterns of *ShCOI1–4*, *ShCOI1–5*, and *ShCOI1–6* in response to smut pathogen infection via RT-qPCR

**Fig. 11:**
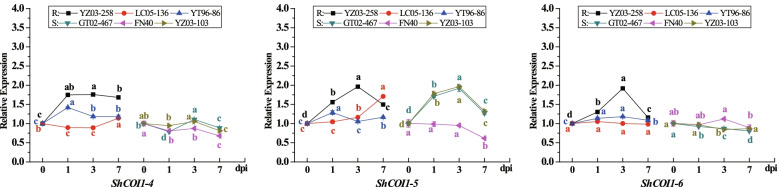
Expression patterns of *ShCOI1–4*, *ShCOI1–5*, and *ShCOI1–6* in the interaction between sugarcane and *Sporisorium scitamineum*. YZ03–258, LC05–136, and YT96–86 were smut-resistant sugarcane cultivars (R); GT02–467, FN40, and YZ03–103 were smut-susceptible sugarcane cultivars (S). Glyceraldehyde-3-phosphate dehydrogenase (*GAPDH*) was used as a reference gene. The 2^−ΔΔCT^ method was applied to obtain the relative expression levels. All data was shown as mean ± standard error (*n* = 3). Different letters representing significant differences were assessed by Duncan test (*p < 0.05)

In the sugarcane-smut pathogen biosystem, the expression patterns of *ShCOI1–4*, *ShCOI1–5*, and *ShCOI1–6* in six *Saccharum* spp. hybrid cultivars (including smut-resistant cultivars YZ03–258, LC05–136, and YT96–86, and smut-susceptible cultivars GT02–467, FN40, and YZ03–103) were evaluated using RT-qPCR (Fig. 11). Compared with the control, the expression levels of *ShCOI1–5* were all significantly increased in three smut-resistant sugarcane cultivars and two smut-susceptible cultivars (GT02–467 and YZ03–103), but were significantly decreased in FN40 at 7 dpi. Except for the increased expression levels of *ShCOI1–4* in LC05–136 at 7 dpi and in GT02–467 at 3 dpi, the expression levels of the *ShCOI1–4* and *ShCOI1–6* genes were both significantly increased in YZ03–258 and YT96–86 and decreased or remained stable in the other four sugarcane cultivars. In summary, *ShCOI1–4*, *ShCOI1–5*, and *ShCOI1–6* can be induced by smut pathogen attack, but their expression patterns vary during the interaction between different sugarcane cultivars and the smut pathogen.

## Discussion

According to previous reports, jasmonic signal molecules can induce plants to activate resistance-related genes and systematically accumulate defense-related proteins to resist biotic and abiotic stresses [7, 8, 49]. As the essential member in the JA signaling pathway, *COI1* has received increasing attention in recent years. It has been reported that the expression levels of *AsCOI1* in *Aquilaria sinensis* can be significantly induced by MeJA, mechanical wounding, and heat [26]. The transcription levels of *HbCOI1* in latex can be induced by JA and tapping [50]. In *O. sativa*, the mutation of *OsCOI1b* can delay leaf senescence, down-regulate several senescence-associated genes (including homologs of *A. thaliana* ETHYLENE INSENSITIVE 3 and ORESARA 1), and result in significant decreases in spikelet fertility and grain filling [18]. When *OsCOI1* was inhibited by RNA interference (RNAi), *O. sativa* plants showed a decrease in resistance to *Cnaphalocrocis medinalis* and activity declines of trypsin protease inhibitor (TrypPI), polyphenol oxidase (PPO), and peroxidase (POD) [25].

Currently, as a polygenic family, the *COI1* gene has been identified and reported in many plants, such as *A. thaliana* [31], *O. sativa* [18], *Artemisia annua* [51], *T. aestivum* [27], and *Hevea brasiliensis* [50]. In this study, a total of 156 COI1 proteins were identified from 19 land plant genomes, including 21 SsCOI1s from *S. spontaneum*, three ShCOI1s from R570, and seven SbCOI1s from *S. bicolor*, but no COI1 was obtained from five algae plants. It is speculated that COI1 may only exist in terrestrial plants because the *COI1* gene family originated after the divergence of the algae and the ancestor of terrestrial plants. Of four groups, only group D in the phylogenetic tree was present in all terrestrial plants, indicating that a common ancestor was shared among the *COI1* gene family from terrestrial plants after the divergence from algae (Fig. 2). In the present study, the numbers of COI1 observed in five eudicots were eight in *M. truncatula* (2n = 4x = 32, autotetraploid) [52]; seven in *A. thaliana* (2n = 2x = 10, diploid) [53], *C. rubella* (2n = 2x = 16, diploid) [54], and *V. vinifera* (2n = 2x = 38, diploid) [55]; and four in *F. vesca* (2n = 2x = 14, diploid) [56] (Fig. 1 and Supplemental Table S1). This result is in conflict with previous reports that the diploid dicots have a single copy of the *COI1* gene, while the polyploid or paleopolyploid dicots may possess a number of *COI1* orthologues in their genomes [21]. In addition, 31 COI1 proteins (three ShCOI1s, seven SbCOI1s, and 21 SsCOI1s) consisted of 434–665 amino acids with MWs ranging from 47.94 to 73.14 kDa, and most of them were predicted as unstable hydrophilic non-secreted proteins (Supplemental Table S3). These sequence characteristics of *ShCOI1s*, *SbCOI1s*, and *SsCOI1s* are common with those in other plants, such as the *TaCOI* gene family [27, 57], *AsCOI1* [26], and *HbCOI1* [50]. Furthermore, ShCOI1, SbCOI1, and SsCOI1 proteins were predicted to be located in the cytoplasm or nucleus (Supplemental Table S3), which was consistent with the subcellular localization of the AsCOI1 [26], SmCOI1 [58], and TaCOI proteins [57]. However, more empirical evidence needed to be provided.

As it shown in phylogenetic tree (Fig. 2), the *COI1* genes from the same lineage, such as mosses, monocots, and eudicots, tended to be clustered to the same clade in group A, group B, and group D, and only COI1 proteins from mosses were clustered in group C. It is speculated that lineage-specific expansion and divergence events have occurred. Interestingly, COI1 proteins from the same species were clustered into different clades. For example, seven SbCOI1 and 21 SsCOI1 proteins were clustered into three groups (A, B, and D) and three ShCOI1s from R570 were clustered into two groups (B and D), revealing that the *COI1* gene family exhibited differences in evolution among species.

According to the phylogenetic tree, protein motifs, and gene structure analysis, those COI1 proteins in the same group showed a similar motif composition and exon/intron structure, but varied among different groups (Fig. 3). For example, most members of *COI1* clustered into group B and group D had two introns, while there were more intron numbers in group A. Furthermore, the number, length, and positions of introns exhibited diversity. Thus, we speculated that the intron loss or gain events occurred during the process of structural evolution of the *COI1* gene family [59, 60]. Motif analysis results revealed the diversity of protein-conserved motifs among the *COI1* gene family, such as the different numbers of motifs 4, 5, 7, 8, and 9 among various COI1 proteins that clustered into different clades. Therefore, the classification and evolution of *COI1* genes might be related to their structural divergence and diversification.

As reported, gene duplication events can provide a primary source of material for the origin of evolutionary novelties, including new gene functions and expression patterns [61]. Chromosomal location, gene type, and gene collinearity analysis are often used to investigate the expansion and evolutionary mechanism of gene families [61, 62]. In this study, 21 *SsCOI1* genes were unevenly distributed among 18 of 32 chromosomes of *S. spontaneum*, and not every *COI1* had homoeologous genes on the homologous chromosomes A, B, C, and D, suggesting that some homologous *COI1* genes may have been lost during the polyploidization of the genome [63]. There was a wide homologous relationship among *S. spontaneum*, R570, and *S. bicolor*, and the Ka/Ks ratios of all duplicated *COI1* genes were < 1, indicating that the *COI1* gene family might have experienced strong purifying selective pressure during evolution (Fig. 4b, c, and Supplemental Table S8). In addition, it was observed that the *COI1* gene family was expanded by various genome duplication events in *S. spontaneum*, *S. bicolor*, and R570 (Fig. 4a and Supplemental Table S7). The different expansion mechanisms demonstrated that the driving forces in the evolution of each *COI1* gene or the *COI1* gene family among different species were diverse, and there may have been functional differentiation among various *COI1* gene family members.

As a key component in the regulation of gene expression, the analysis of *cis*-acting regulatory elements in gene promoters can assist to elaborate the regulation and function of individual genes and their interaction with other genes [64, 65]. In the present study, a large number of promoter core elements were identified in the promoter sequences of *ShCOI1s*, *SbCOI1s*, and *SsCOI1s* that were involved in stress responsiveness (such as drought, low-temperature, and wound stress), hormone responsiveness (like SA, ABA, and MeJA), light responsiveness, and growth and development (Fig. 5 and Supplemental Table S9). This finding was similar to that observed in the promoter regions of *HbCOI1* [50] and *TaCOI* genes [57]. The existence of these functional elements indicates that *COI1* genes may play a role in sugarcane development and defense against various environmental stresses via participating in different regulatory mechanisms.

Gene expression patterns are usually related to their function [66]. As reported, the expression levels of *COI1* genes in different plants are spatiotemporal [26, 50, 57, 58]. For instance, in *H. brasiliensis*, *HbCOI1* has high transcription levels in laticifers, but low levels in bark and leaf tissues [50]. In *Solanum melongena*, the transcription levels of *SmCOI1* are significantly down-regulated in anther indehiscence, which is related to the normal development of anthers [58]. In *A. sinensis*, the *AsCOI1* gene was highly expressed in roots and stems, the two major organs of agarwood formation [26]. In addition, the members of the *TaCOI* gene family are expressed differently in various tissues, with the higher expression levels in stem, leaf, petal, pistil, stamen, and glume tissues than in roots [57]. Analogously, *COI1* genes were constitutively expressed in sugarcane cultivar ROC22, but their expression patterns were diverse in different tissues (Fig. 6). For example, the expression levels of the group B genes (*SsCOI1–1a*, *−1b*, *−3a*, *−3b*, and *−3c*) were the highest in the bud and the lowest in the leaf. Compared with the other group genes, *SsCOI1* genes, which clustered into group D, showed abundant transcripts and had the highest expression levels in stem pith. These results indicate that *COI1* genes exhibit a tissue-specific pattern, and the same expression pattern suggests a similar function in the growth and development process.

RNA-seq data revealed that the *COI1* gene family played a positive role in sugarcane response to drought and cold stresses with different expression patterns (Fig. 7). Interestingly, this finding is consistent with the prediction that the *COI1* promoter sequence contains a large number of drought and low temperature response elements. To validate the function diversities of sugarcane *COI1* genes, the expression levels of *SsCOI1s* under MeJA treatment were assessed by RT-qPCR (Fig. 9). The results show that various *SsCOI1* genes may play different roles in response to MeJA. To further elucidate the functions of the *COI1* gene family in a sugarcane cultivar, three *ShCOI1* genes homologous with *SsCOI1–4b*, *SsCOI1–1b*, and *SsCOI1–3b* were cloned from ROC22. RT-qPCR results revealed that the transcription of *ShCOI1–4* was decreased under cold (4 °C) and drought (PEG) stresses, while it was up-regulated significantly after SA and ABA treatments. The expression levels of *ShCOI1–5* were up-regulated remarkably under SA and drought stresses, and down-regulated under ABA stress, and remained unchanged under cold stress. The transcriptions of the *ShCOI1–6* gene were increased under SA, ABA, and drought stresses, but remained unchanged under cold stress (Fig. 10). These results were consistent with previous reports that *COI1* was involved in the response of plants to various abiotic stresses and exhibited functional divergence [21, 57]. Likewise, in the *TaCOI* gene family, *TaCOI2* (*TaCOI2-A* and *TaCOI2-B*) and *TaCOI6* (*TaCOI6-A*, *TaCOI6-B*, and *TaCOI6-D*) were clustered into group A, while *TaCOI5* (*TaCOI5-A*, *TaCOI5-B*, and *TaCOI5-D*) was clustered into group D [57]. The expression levels of *TaCOI2* were up-regulated under the ABA, GA, and low-temperature treatments, but down-regulated under the IAA and MeJA treatments [57]. The transcripts of *TaCOI6* could be induced by ABA and MeJA, but were suppressed by IAA and PEG [57]. The transcription levels of *TaCOI5* were increased under the GA, low temperature, and PEG treatments, while they were decreased after the ABA, IAA, MeJA, and salinity treatments [57]. In *Z. mays*, the expression levels of four *ZmCOIs* (clustered into group A) responded to plant hormones were detected, and the results showed that *ZmCOI1a* and *ZmCOI1b* were strongly induced by JA and ABA, while *ZmCOI1c* and *ZmCOI2* were less-expressed in maize tissues and slightly induced by JA and ABA, but there was no significant induction for *ZmCOI1a*, *ZmCOI1b*, *ZmCOI1c* and *ZmCOI2* by 1-Aminocyclopropane-1-carboxylic acid, GA, 1-Naphthylacetic acid, and SA [21]. In addition, the restoration of male fertility in *Arabidopsis* mutant *coi1–1* could result in plants that overexpressed *ZmCOI1a*, *ZmCOI1b*, or *ZmCOI1c*, but not *ZmCOI2*, indicating the successful complementation of *coi1–1* sterility by *ZmCOI1a*, *ZmCOI1b*, and *ZmCOI1c* and the functional divergence of *ZmCOIs* [21]. It should be stressed that *COI1* genes may exhibit inconsistent expression patterns under certain environmental stresses even if they are clustered into the same group.

It has been reported that JA and SA are mostly related to plant resistance to pathogen infection [11, 46]. Therefore, the *COI1* gene family may be involved in sugarcane response to pathogen infection. Transcriptome analysis showed that *SsCOI1* genes could be induced during the interaction between sugarcane and the smut pathogen, and the alleles showed similar expression patterns. Among them, the expression levels of *SsCOI1–4b* and *SsCOI1–3b* were up-regulated in smut-resistant cultivar YC05–179, but down-regulated in smut-susceptible cultivar ROC22, while *SsCOI1–1b* was down-regulated in both YC05–179 and ROC22. Furthermore, the expression patterns of *ShCOI1–4*, *ShCOI1–5*, and *ShCOI1–6*, the homologous genes of *SsCOI1–4b*, *SsCOI1–1b*, and *SsCOI1–3b*, respectively, were different in the interaction between the six different sugarcane cultivars and the smut pathogen (Fig. 11). Similarly, *TaCOI1* took part in the early defense of compatible and incompatible wheat responses to *Blumeria graminis* (*Bgt*), and the response time was earlier in the resistant cultivars than in the susceptible ones [27]. Using virus-induced gene silencing, the expression of *TaCOI1* decreased significantly, and the rate of successful penetration by *Bgt* was higher than that of the control. This indicates that *TaCOI1* may play a key role in wheat-*Bgt* interactions [27]. In *A. thaliana*, two mutant alleles of *coil* conferred hypersusceptibility to the necrotrophic pathogen *Sclerotinia sclerotiorum* than wild-type or heterozygous plants [67]. Furthermore, overexpressed *ZmCOIs* in the *Arabidopsis coi1–1* mutant plants can cause the restoration of resistance to the leaf pathogen *Botrytis cinerea* and the soil-borne pathogen *Pythium aristosporum* [21]. Taking the above findings into consideration, we conclude that *COI1* genes have multiple functions and participate in sugarcane defense against various environmental stresses via different regulatory mechanisms.

## Conclusion

A total of 156 COI1s, including 21 SsCOI1s, seven SbCOI1s, and three ShCOI1s, were identified from 19 species and could be clustered into four groups. The analysis of *cis*-acting elements, tissue-specific expression, and expression profiles under various stresses suggests that *COI1* genes participate in growth, development, and response to various stresses in sugarcane. Furthermore, three *COI1* genes*, ShCOI1–4*, *ShCOI1–5*, and *ShCOI1–6*, were obtained by homologous cloning in the sugarcane cultivar ROC22 and could be induced by the stresses of drought, cold, ABA, SA, and *S. scitamineum* with divergent expression profiles. The results illustrate the fact that sugarcane *COI1* genes may actively respond to biotic and abiotic stresses via different regulatory mechanisms. The present study laid a foundation for the functional identification of sugarcane *COI1* genes and provided a theoretical basis for molecular breeding of sugarcane resistance.

## Materials and methods

### Plant materials

Eight *Saccharum* spp. hybrid cultivars (including YT96–86, LC05–136, YZ03–258, ROC22, GT02–467, YZ03–103, FN40, and YC05–179) and smut whip were obtained from the Key Laboratory of Sugarcane Biology and Genetic Breeding, Ministry of Agriculture and Rural Affairs (Fuzhou, China).

The root, stem pith, leaf ^+ 1^, bud, and epidermis tissues of nine consistent 10-month-old ROC22 plants (the prevalent sugarcane cultivar in mainland China) were collected [42]. Four-month-old hydroponic ROC22 tissue-cultured plantlets were sprayed with 100 mM ABA, 5 mM SA (containing 0.01% Tween-20, *v/v*), 100 μM MeJA (containing 0.1% ethanol and 0.05% Tween-20, *v/v*), and 25% PEG 8000 at 28 °C with 16 h light and 8 h darkness [42, 68]. The leaves under SA and MeJA treatments were harvested at 0, 3, 12, and 24 h, the leaves under ABA treatment were collected at 0, 3, 6, and 12 h, and the leaves under PEG stress were harvested at 0, 0.5, 3, 6, and 24 h [42, 68]. For cold stress, the whole ROC22 plantlets were kept at a low temperature of 4 °C with 16 h light and 8 h darkness for 0, 6, 12, 24, and 48 h [42, 68].

The stems of eight 10-month-old sugarcane cultivars, including smut-resistant cultivars YC05–179, YZ03–258, LC05–136, and YT96–86; and smut-susceptible cultivars GT02–467, ROC22, FN40, and YZ03–103, were cut into two-bud sets, immersed for 1 day in flowing water, and cultivated under a light–dark regime (16 h of light and 8 h of darkness) at 32 °C until the germinating seedlings with a bud height of about 2 cm [44, 69]. Then, the bud was inoculated with 5 × 10^6^ spores·mL^− 1^ *S. scitamineum* (0.01% Tween-20, *v/v*), and the control group was inoculated with sterile water (0.01% Tween-20, *v*/*v*) [43, 44]. All the materials were cultivated at 28 °C with a photoperiod of 16 h light and 8 h darkness [43, 44]. Five buds of YZ03–258, LC05–136, YT96–86, GT02–467, FN40, and YZ03–103 at 0, 1, 3, and 7 dpi were harvested and immediately frozen in liquid nitrogen for gene expression analysis [44, 69]. Five buds of YC05–179 and ROC22 were collected at 0, 1, 2, and 5 d after *S. scitamineum* inoculation for RNA-seq [70].

Each treatment included three biological replicates. All samples were immediately frozen in liquid nitrogen and stored at − 80 °C.

### RNA extraction and first-strand cDNA synthesis

Total RNA was extracted from the collected samples using TRIzol™ (Invitrogen, Carlsbad, USA). RNA (1.0 μg) was reverse transcribed to the first-strand cDNA using a Prime-Script™ RT Reagent Kit (TaKaRa, Dalian, China) for RT-qPCR analysis. The cDNA used as cloning templates was synthesized from the RNA of ROC22 buds using a HiScript II 1st Strand cDNA Synthesis Kit (Vazyme, Nanjing, China).

### Identification of the *COI1* gene family

To identify the *COI1* gene family, the genomic data of a total of 24 species were collected (Supplemental Table S10). The genomic data of 19 plants (*A. thaliana*, *A. trichopoda*, *A. comosus*, *B. distachyon*, *C. rubella*, *F. vesca*, *M. truncatula*, *O. sativa*, *P. hallii*, *P. patens*, R570, *S. italica*, *S. bicolor*, *S. spontaneum*, *S. fallax*, *S. moellendorffii*, *T. aestivum*, *V. vinifera*, *Z. mays, C. subellipsoidea* C169, and *M. pusilla* CCMP1545) were downloaded from Phytozome (https://phytozome.jgi.doe.gov/pz/portal.html). The genomic data of *C. crispus*, *C. merolae*, and *G. sulphuraria* were downloaded from Ensembl (http://plants.ensembl.org/index.html). The genomic data of *S. spontaneum* was downloaded from the link of http://www.life.illinois.edu/ming/ downloads/Spontaneum_genome/ [36]. The monoploid reference genome of R570 was obtained from the Sugarcane Genome Hub (http://sugarcane-genome.cirad.fr/) [38]. Two Hidden Markov Model (HMM) profiles (PF18511.1 and PF18791.1), which were predicted by Pfam (http://pfam.xfam.org/search#tabview=tab1), were download from HMMER (https://www.ebi.ac.uk/Tools/hmmer) and used for the HMMER search [71]. Hmmsearch (HMMER package version 3.1b2) was used to search candidate COI1s from the genomic data of 24 species [71]. All obtained sequences were input into the Conserved Domain Database (CDD) (https://www.ncbi.nlm.nih.gov/cdd) to search the protein domain [72]. The *COI1* gene family members were confirmed after removing incomplete sequences. Allele genes were designated as the same name followed by the letters “a,” “b,” “c,” and “d”, and duplicated genes were designated as the same name followed by the letter “e” in *S. spontaneum*. The *COI1* genes in *T. aestivum* and *Z. mays* were named according the research of Bai et al. [57] and An et al. [21], respectively.

### Sequence characteristics of the *COI1* gene family

All the identified *COI1* genes in *S. bicolor*, R570, and *S. spontaneum* were submitted to ExPASy (http://web.expasy.org/protparam/) to analyze their amino acid numbers, MW, theoretical *p*I, instability index, and GRAVY. All full-length proteins were submitted to SOPMA (https://npsa-prabi.ibcp.fr/cgi-bin/npsa_automat.pl?page=npsa_sopma.html) for secondary structure analysis. The predictions of signal peptides, transmembrane structures, and subcellular localizations were conducted by SignalP-5.0 (http://www.cbs.dtu.dk/services/SignalP/), TMHMM (http://www.cbs.dtu.dk/services/TMHMM/), and Plant-mPloc (http://www.csbio.sjtu.edu.cn/bioinf/euk-multi-2/), respectively. In addition, the percent identity matrixes between COI1 proteins in *S. bicolor*, R570, *S. spontaneum*, and sugarcane hybrid cultivar ROC22 were calculated using DNAMAN.

### Multiple sequence alignment and phylogenetic analysis

Multiple sequence alignment (MSA) of COI1 proteins was conducted by ClustalW in MEGA 6.60 with default parameters. Three phylogenetic trees in this study, including one phylogenetic tree of the 156 COI1 proteins from 19 plant species, one phylogenetic tree of 21 SsCOI1, seven SbCOI1, and three ShCOI1 proteins, and one phylogenetic tree of 21 SsCOI1s, were constructed using the maximum likelihood method (JTT + G model, complete deletion, and 1000 bootstrap replicates) based on the above alignments [73]. Evolview (https://evolgenius.info/evolview-v2/#mytrees/SHOWCASES/showcase%2002) [74] was used to display and edit the phylogenetic tree.

### Motif and gene structure analysis of *SbCOI1s*, *ShCOI1s*, and *SsCOI1s*

Amino acid sequences of SbCOI1s, ShCOI1s, and SsCOI1s were submitted to the Multiple Em for Motif Elicitation online program (http://meme-suite.org/tools/meme) to identify the conserved motifs [75]. The parameters were as follows: maximum motif number, 10; maximum motif width, 50; minimum motif width, 6; and distribution of motif occurrences with zero or one per sequence. Diagrams of exon-intron structures were drawn using the Gene Structure Display Server 2.0 (http://gsds.gao-lab.org/). TBtools (Toolbox for Biologists) v1.09832 and Adobe Illustrator CS6 were used to display and edit the phylogenetic tree, conserved motifs, and gene structures [76].

### Chromosomal locations and collinearity analysis of *SbCOI1s*, *ShCOI1s*, and *SsCOI1s*

The physical locations of *COI1s* on the chromosomes of *S. bicolor*, R570, and *S. spontaneum* were analyzed using MapGene2Chrom (MG2C) software (http://mg2c.iask. in/mg2c_v2.1/). Multiple Collinearity Scan toolkit (MCScanX) and TBtools were used with the default parameters to analyze the synteny block and gene duplication pattern [76, 77]. The values of Ka/Ks between orthologous gene pairs were calculated by TBtools to study the selection pressure acting on the evolution of the *COI1* gene family [76].

### *Cis*-acting regulatory element analysis in the promoter regions of *COI1* genes

A 2000 bp sequence upstream of the start site of gene translation of *SbCOI1s*, *ShCOI1s*, and *SsCOI1s* was retrieved from genomic data as the promoter sequence, and its *cis*-regulatory elements were predicted using the PlantCARE online program (http://bioinformatics.psb.ugent.be/webtools/plantcare/html/) [78]. The results of the prediction were visualized using TBtools [76].

### Expression profiles of *SsCOI1s* in sugarcane based on RNA-seq

The RNA of the roots, stem piths, leaves, buds, and epidermis in ROC22 and the buds of YC05–179 and ROC22 inoculated with *S. scitamineum* for 0, 1, 2, and 5 d [70] were sequenced and assembled by the Biomarker Technologies Company limited (Beijing, China). The original data were obtained by Illumina technology, and after passing quality control, the data were analyzed using the *S. spontaneum* genome as the reference annotation library. The fragments per kilobase of transcript per million mapped (FPKM) was used as an indicator to measure the expression levels of transcripts or genes. For drought and cold treatments, the original data (PRJNA590595 and PRJNA636260) were downloaded from the Sequence Read Archive database (https://www.ncbi.nlm.nih.gov/sra/). The leaves of *Saccharum* hybrid cultivar Co 8021 under drought treatment were harvested at 0, 2, 6, and 10 d, and water-recovered at 10 d. The leaves of *S. spontaneum* under cold treatment were collected at 0, 0.5, 1, and 6 h. The sequence quality of these data was improved by Fastp [79]. The Hisat2 program was used to map sequence data to the *S. spontaneum* genome [80]. The count read and normalization of the data were conducted by the featurCounts in the Subread package and the trimmed mean of M-values (TMM) [81, 82]. The gene IDs of these transcriptomes followed the format of the original gene ID of *S. spontaneum*, which was relevant to the related search of their homologous genes. The expression levels of *SsCOI1s* in different sugarcane tissues and in response to drought, cold, and *S. scitamineum* stresses were mined from these RNA-seq data. The heat map showing the log_2_ (FPKM or TMM) expression profiles was generated by TBtools [76].

### Cloning and sequences analysis of candidate *S. spontaneum SsCOI1* genes in sugarcane cultivar

According to the sequences of *SsCOI1s*, the specific primers for *ShCOI1–4*, *ShCOI1–5*, and *ShCOI1–6* (Supplemental Table S11) which were clustered into two different groups of *COI1* gene family and with different expression patterns in response to MeJA were designed using Primer premier 5.0 software. The cDNA of ROC22 bud was used as a template for gene cloning. The transcription-polymerase chain reaction (RT-PCR) system contained 1.0 μL cDNA template, 1.0 μL each of the forward and reverse primers (10 μM), 12.5 μL 2× Phanta Max buffer, 0.5 μL dNTPs (2.5 mM), and 0.5 μL Phanta Max Super-Fidelity DNA Polymerase (Vazyme, Nanjing, China), and 8.5 μL ddH_2_O. The PCR reaction conditions were as follows: 95 °C for 3 min; 35 cycles of 95 °C for 15 s, 56 °C for 15 s, and 72 °C for 2 min 30 s; and 72 °C for 5 min. PCR products were gel-purified, cloned into pMD19-T vector (TaKaRa, Dalian, China), and sequenced [42]. Amino acid sequence alignment among *ShCOI1–4*, *ShCOI1–5*, *ShCOI1–6*, and ZmCOIs (ZmCOI1a, GRMZM2G125411; ZmCOI1b, GRMZM2G151536; ZmCOI1c, GRMZM2G353209; ZmCOI2, GRMZM2G079112) was performed by NTI software [21, 31, 45].

### RT-qPCR analysis

Seven primer pairs of non-allelic *SsCOI1* genes were designed by Beacon Designer 8.0 (Supplemental Table S11). Due to the high amino acid sequences similarity (Supplemental Table S5), the primer pairs of *SsCOI1–4*, *SsCOI1–1*, *SsCOI1–3* used in RT-qPCR analysis were the same as those of *ShCOI1–4*, *ShCOI1–5*, and *ShCOI1–6*, respectively (Supplemental Table S5 and Supplemental Table S11). The expression levels of the seven *SsCOI1* genes under MeJA stress and those of *ShCOI1–4*, *ShCOI1–5*, and *ShCOI1–6* in the six sugarcane cultivars (YZ03–258, LC05–136, YT96–86, GT02–467, FN40, and YZ03–103) infected by smut pathogen and under hormones and abiotic stresses (ABA, SA, cold, and PEG) were detected using RT-qPCR. The RT-qPCR was performed on Applied biosystems Q3 (ThermoFisher, Waltham, USA) system using the SYBR-green dye method with the conditions of 50 °C for 2 min; 95 °C for 10 min; 40 cycles of 95 °C for 15 s and 60 °C for 1 min. The total volume of the RT-qPCR reaction system was 20 μL, which included 10 μL of the 2 × ChamQ Universal SYBR qPCR Master Mix, 0.4 μL of the primer (10 μM), 1.0 μL of the template (10 × cDNA diluted liquid), and 8.2 μL ddH_2_O. Glyceraldehyde-3-phosphate dehydrogenase (*GAPDH*) (Supplemental Table S11) was used as a reference gene [83]. Each sample was assessed using three replicates. Expression levels of *COI1* genes were calculated using the 2^−∆∆CT^ algorithm [84]. Significant differences (*p < 0.05) and standard error (SE) were determined by the Duncan’s new multiple range test by using Data Processing System v9.50 software, and the histogram was graphed by Origin 9.0.

## Supplementary Information

**Additional file 1: Figure S1**. Amino acid sequence alignment of ShCOI1s and ZmCOIs. *Zea mays* COI1s: ZmCOI1a (GRMZM2G125411), ZmCOI1b (GRMZM2G151536), ZmCOI1c (GRMZM2G353209), and ZmCOI2 (GRMZM2G079112). F-box domains were underlined with the black line. Transp_inhibit (transport inhibitor response 1 protein) domains were underlined with the red line. AMN1 domains (leucine-rich repeat (LRR) protein) were underlined with the blue line. Asterisks indicated the binding sites of coronatine/JA-Ile in the COI1-JAZ complex. Plus signs indicated conserved amino acid residues of F-box domains. Site 1, Site 2, Site 3, and Site 4 indicated four JAZ-binding sites involved in the COI1-JAZ interaction.

**Additional file 2: Figure S2**. Chromosomal distribution of the *COI1* gene family in *Sorghum bicolor*, R570, and *Saccharum spontaneum.* (a) The *S. bicolor* chromosome. (b) The *S. spontaneum* chromosome. (c) The *Saccharum* spp. hybrid cultivar R570 chromosome. *SbCOI1*, *ShCOI1*, and *SsCOI1* represented the *COI1* gene in *S. bicolor*, R570, and *S. spontaneum*. The scale bar on the left indicated the chromosome length (megabasee, Mb). The name of each chromosome showed on the top of each chromosome.

**Additional file 3: Table S1**. Identification of* COI1* genes in different plants. **Table S2**. Conserved amino acid residues of COI1 proteins among different groups. **Table S3**. Characterization of *SbCOI1*, *ShCOI1*, and *SsCOI1* genes. **Table S4**. Secondary structure analysis of SbCOI1, ShCOI1, and SsCOI1 proteins. **Table S5**. Percentage of identity between 7 SbCOI1s, 6 ShCOI1s, and 21 SsCOI1s was calculated using DNAMAN software. **Table S6**. Ten conserved motifs predicted in the COI1 proteins. **Table S7**. The gene type of *COI1* genes in *Sorghum bicolor*, *Saccharum* spp. hybrid cultivar R570, and *Saccharum spontaneum*. **Table S8**. Syntenic relationships and selection pressure among *Sorghum bicolor*, *Saccharum* spp. hybrid cultivar R570, and *Saccharum spontaneum*. **Table S9**. Promoter *cis*-regulatory elements analysis of *SbCOI1*, *ShCOI1*, and *SsCOI1* genes. **Table S10**. Sources of *COI1* genes from sequenced species included in this study. **Table S11** Primers used in this study.

## Data Availability

The data supporting the conclusions of this article are within the paper.

## Abbreviations

*COI1*: Coronatine insensitive 1
JA: Jasmonic acid
SCF: SKP1 + Cdc53/cullin+Rbx1 + F-box
JAZ: Jasmonate ZIM-domain
MYC2: Myelocytomatosis2
JA-Ile: Jasmonoyl-l-isoleucine
JAR1: Jasmonic acid-amido synthetase
LRR: Leucine-rich repeats
RCA: Rubisco activase
RT-qPCR: Real-time quantitative PCR
MeJA: Methyl jasmonate
SA: Salicylic acid
ABA: Abscisic acid
MWs: Molecular weights
*p*I: isoelectric point
GRAVY: Grand average of hydropathicity
UTR: Untranslated regions
WGD: Whole-genome duplication
Ka/Ks: Nonsynonymous/synonymous
IAA: Auxin
GA: Gibberellin
RNA-seq: Transcriptome sequencing
FPKM: Fragments per kilobase of transcript per million mapped
TMM: Trimmed mean of M-values
dpi: day (s) post-inoculation
RT-PCR: Transcription-polymerase chain reaction
PEG: Polyethylene glycol
*GAPDH*: Glyceraldehyde-3-phosphate dehydrogenase
SE: Standard error
RNAi: RNA interference
TrypPI: Trypsin protease inhibitor
PPO: Polyphenol oxidase
POD: Peroxidase
*Bgt*: *Blumeria graminis*
HMM: Hidden Markov Model
CDD: Conserved Domain Database
MSA: Multiple sequence alignment
TBtools: Toolbox for Biologists
MG2C: MapGene2Chrom
MCScanX: Multiple Collinearity Scan toolkit
