# Supplementary material for: Genome-wide identification and expression analysis of the coronatine-insensitive 1 (COI1) gene family in response to biotic and abiotic stresses in Saccharum
Source: BMC Genomics. 2022 Jan 8;23:38. doi: 10.1186/s12864-021-08255-0 (PMC8742417; doi:10.1186/s12864-021-08255-0)
Supplement: Supplementary file 1 — Additional file 1: Figure S1. Amino acid sequence alignment of ShCOI1s and ZmCOIs. Zea mays COI1s: ZmCOI1a (GRMZM2G125411), ZmCOI1b (GRMZM2G151536), ZmCOI1c (GRMZM2G353209), and ZmCOI2 (GRMZM2G079112). F-box domains were underlined with the black line. Transp_inhibit (transport inhibitor response 1 protein) domains were underlined with the red line. AMN1 domains (leucine-rich repeat (LRR) protein) were underlined with the blue line. Asterisks indicated the binding sites of coronatine/JA-Ile in the COI1-JAZ complex. Plus signs indicated conserved amino acid residues of F-box domains. Site 1, Site 2, Site 3, and Site 4 indicated four JAZ-binding sites involved in the COI1-JAZ interaction. [file 12864_2021_8255_MOESM1_ESM.docx]

**Genome-wide identification and expression analysis of the coronatine-insensitive 1 (*COI1*) gene family in response to biotic and abiotic stresses in *Saccharum***

Tingting Sun^1^

E-mail address: [sunting3221@163.com](mailto:sunting3221@163.com)

Yintian Meng^1^

E-mail address: [mengyintian8927@163.com](mailto:mengyintian8927@163.com)

Guangli Cen^1^

E-mail address: [cgl33579@163.com](mailto:cgl33579@163.com)

Aoyin Feng^1^

E-mail address: [feng_aoyin98@163.com](mailto:feng_aoyin98@163.com)

Weihua Su^1^

E-mail address: [suweihua2016@126.com](mailto:suweihua2016@126.com)

Yanling Chen^1^

E-mail address: [chenyanling1218@163.com](mailto:chenyanling1218@163.com)

Chuihuai You^2^

E-mail address: [you123chui@163.com](mailto:you123chui@163.com)

Youxiong Que^1,3,^*

E-mail address: [queyouxiong@126.com](mailto:queyouxiong@126.com)

Yachun Su^1,3,^*

E-mail address: [syc2009mail@163.com](mailto:syc2009mail@163.com)

^1^ Key Laboratory of Sugarcane Biology and Genetic Breeding, Ministry of Agriculture and Rural Affairs, College of Agriculture, Fujian Agriculture and Forestry University, Fuzhou, 350002, Fujian, China

^2^ College of Life Sciences, Fujian Agriculture and Forestry University, Fuzhou, 350002, Fujian, China

^3^ Key Laboratory of Genetics, Breeding and Multiple Utilization of Crops, Ministry of Education, College of Agriculture, Fujian Agriculture and Forestry University, Fuzhou, 350002, Fujian, China

***Corresponding should be addressed to** [queyouxiong@126.com](mailto:queyouxiong@126.com) (Y. Que) and [syc2009mail@163.com](mailto:syc2009mail@163.com) (Y. Su).

**The full postal address of the submitting author Youxiong Que is as follows:** Key Laboratory of Sugarcane Biology and Genetic Breeding, Ministry of Agriculture and Rural Affairs, College of Agriculture, Fujian Agriculture and Forestry University, Fuzhou, 350002, Fujian, China.


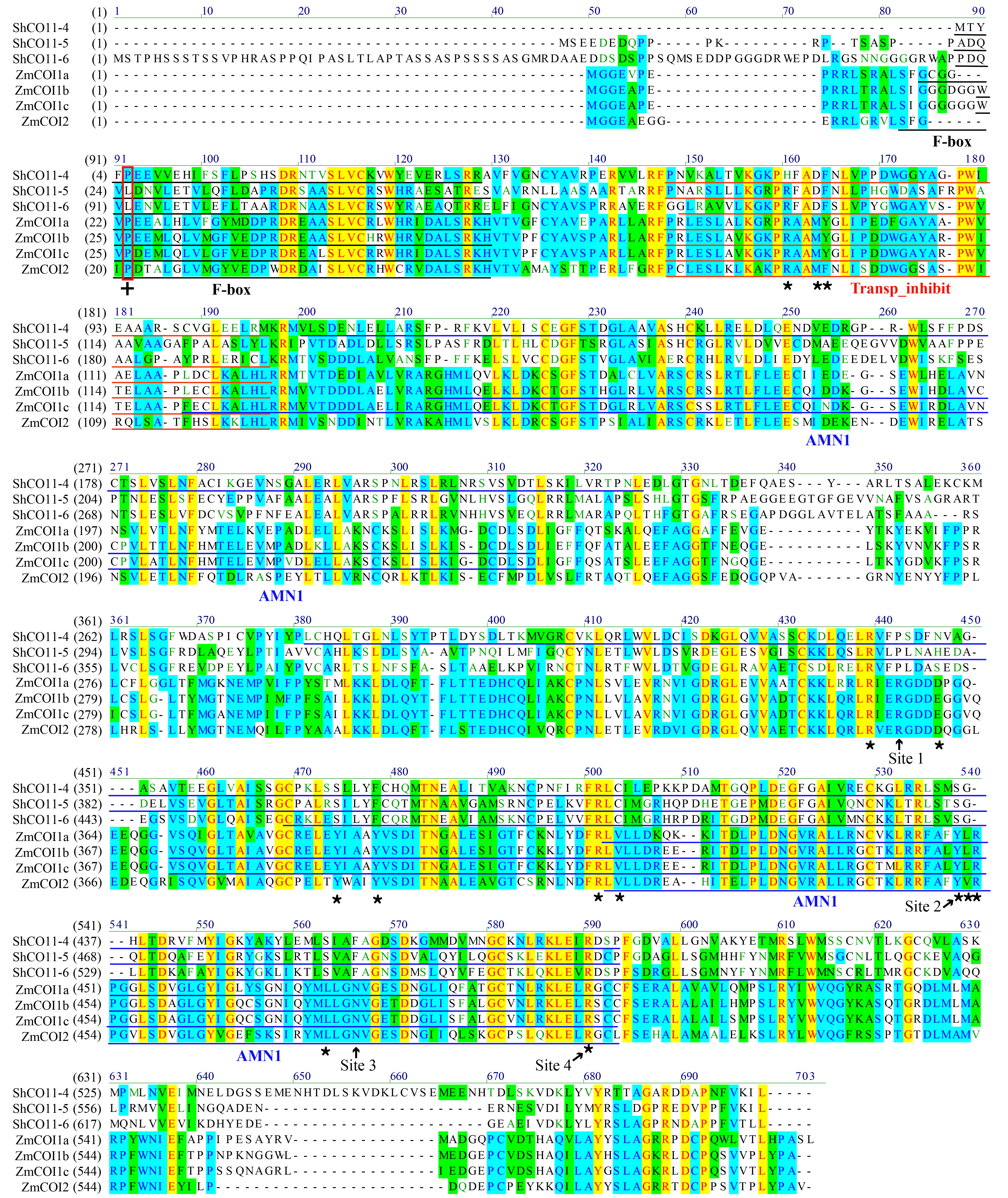


**Figure S1** Amino acid sequence alignment of ShCOI1s and ZmCOIs. *Zea mays* COI1s: ZmCOI1a (GRMZM2G125411), ZmCOI1b (GRMZM2G151536), ZmCOI1c (GRMZM2G353209), and ZmCOI2 (GRMZM2G079112). F-box domains were underlined with the black line. Transp_inhibit (transport inhibitor response 1 protein) domains were underlined with the red line. AMN1 domains (leucine-rich repeat (LRR) protein) were underlined with the blue line. Asterisks indicated the binding sites of coronatine/JA-Ile in the COI1-JAZ complex. Plus signs indicated conserved amino acid residues of F-box domains. Site 1, Site 2, Site 3, and Site 4 indicated four JAZ-binding sites involved in the COI1-JAZ interaction.
