# Supplementary material for: Genome-wide identification and expression analysis of the coronatine-insensitive 1 (COI1) gene family in response to biotic and abiotic stresses in Saccharum
Source: BMC Genomics. 2022 Jan 8;23:38. doi: 10.1186/s12864-021-08255-0 (PMC8742417; doi:10.1186/s12864-021-08255-0)
Supplement: Supplementary file 2 — Additional file 2: Figure S2. Chromosomal distribution of the COI1 gene family in Sorghum bicolor, R570, and Saccharum spontaneum. (a) The S. bicolor chromosome. (b) The S. spontaneum chromosome. (c) The Saccharum spp. hybrid cultivar R570 chromosome. SbCOI1, ShCOI1, and SsCOI1 represented the COI1 gene in S. bicolor, R570, and S. spontaneum. The scale bar on the left indicated the chromosome length (megabasee, Mb). The name of each chromosome showed on the top of each chromosome. [file 12864_2021_8255_MOESM2_ESM.docx]

**Genome-wide identification and expression analysis of the coronatine-insensitive 1 (*COI1*) gene family in response to biotic and abiotic stresses in *Saccharum***

Tingting Sun^1^

E-mail address: [sunting3221@163.com](mailto:sunting3221@163.com)

Yintian Meng^1^

E-mail address: [mengyintian8927@163.com](mailto:mengyintian8927@163.com)

Guangli Cen^1^

E-mail address: [cgl33579@163.com](mailto:cgl33579@163.com)

Aoyin Feng^1^

E-mail address: [feng_aoyin98@163.com](mailto:feng_aoyin98@163.com)

Weihua Su^1^

E-mail address: [suweihua2016@126.com](mailto:suweihua2016@126.com)

Yanling Chen^1^

E-mail address: [chenyanling1218@163.com](mailto:chenyanling1218@163.com)

Chuihuai You^2^

E-mail address: [you123chui@163.com](mailto:you123chui@163.com)

Youxiong Que^1,3,^*

E-mail address: [queyouxiong@126.com](mailto:queyouxiong@126.com)

Yachun Su^1,3,^*

E-mail address: [syc2009mail@163.com](mailto:syc2009mail@163.com)

^1^ Key Laboratory of Sugarcane Biology and Genetic Breeding, Ministry of Agriculture and Rural Affairs, College of Agriculture, Fujian Agriculture and Forestry University, Fuzhou, 350002, Fujian, China

^2^ College of Life Sciences, Fujian Agriculture and Forestry University, Fuzhou, 350002, Fujian, China

^3^ Key Laboratory of Genetics, Breeding and Multiple Utilization of Crops, Ministry of Education, College of Agriculture, Fujian Agriculture and Forestry University, Fuzhou, 350002, Fujian, China

***Corresponding should be addressed to** [queyouxiong@126.com](mailto:queyouxiong@126.com) (Y. Que) and [syc2009mail@163.com](mailto:syc2009mail@163.com) (Y. Su).

**The full postal address of the submitting author Youxiong Que is as follows:** Key Laboratory of Sugarcane Biology and Genetic Breeding, Ministry of Agriculture and Rural Affairs, College of Agriculture, Fujian Agriculture and Forestry University, Fuzhou, 350002, Fujian, China.


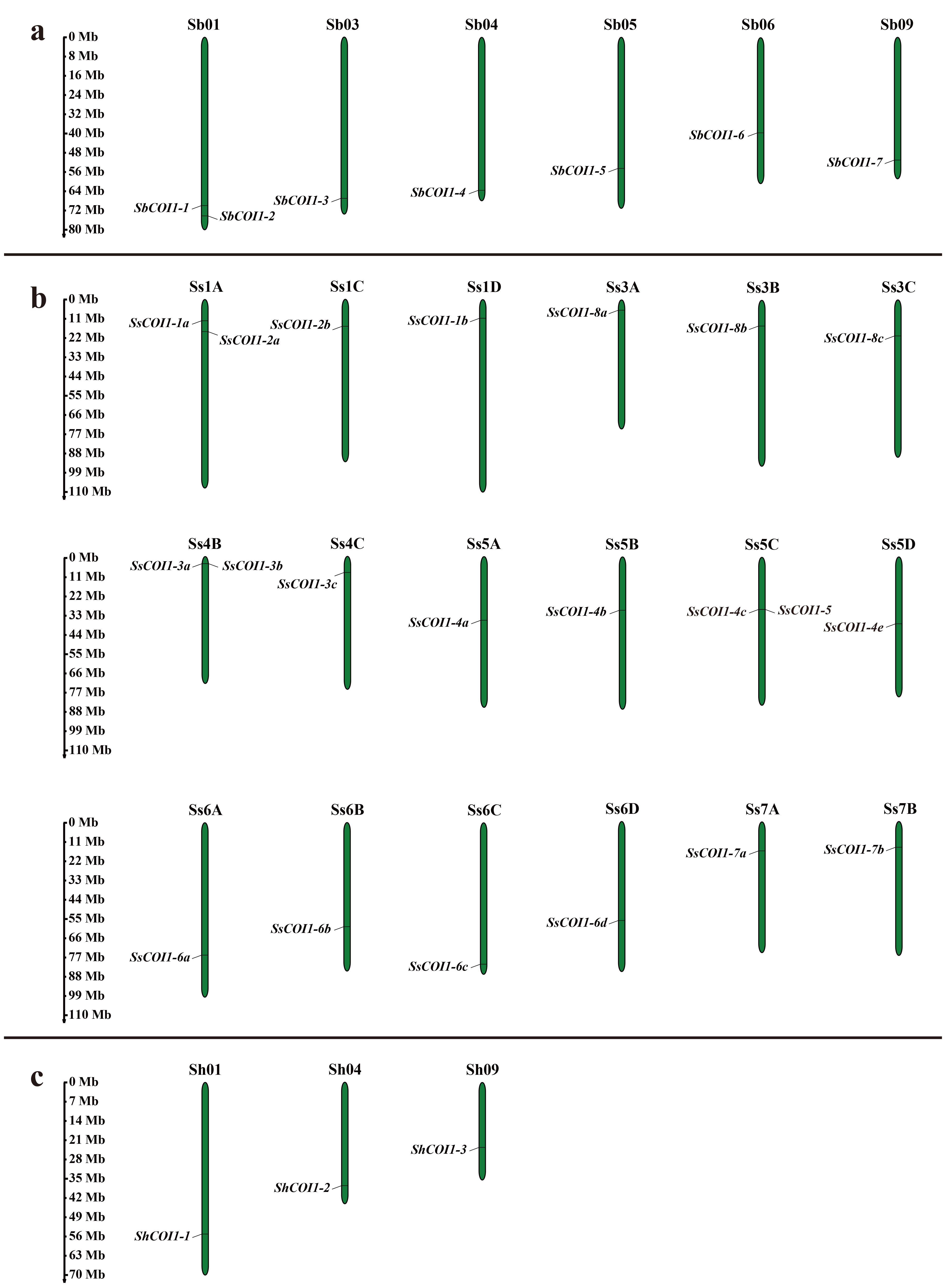


**Figure S2** Chromosomal distribution of the *COI1* gene family in *Sorghum bicolor*, R570, and *Saccharum spontaneum.* **(a)** The *S. bicolor* chromosome. **(b)** The *S. spontaneum* chromosome. **(c)** The *Saccharum* spp. hybrid cultivar R570 chromosome. *SbCOI1*, *ShCOI1*, and *SsCOI1* represented the *COI1* gene in *S. bicolor*, R570, and *S. spontaneum*. The scale bar on the left indicated the chromosome length (megabasee, Mb). The name of each chromosome showed on the top of each chromosome.
